# Statin exposure and risk of colorectal cancer in patients with inflammatory bowel disease: a systematic review and meta-analysis

**DOI:** 10.3389/fmed.2024.1507739

**Published:** 2024-11-22

**Authors:** Ai-juan Li, Hai-yin Jiang, Yong-hui Jia

**Affiliations:** ^1^Pharmacy Department, The 960th Hospital of PLA, Jinan, China; ^2^State Key Laboratory for Diagnosis and Treatment of Infectious Diseases, Collaborative Innovation Center for Diagnosis and Treatment of Infectious Diseases, The First Affiliated Hospital, College of Medicine, Zhejiang University, Hangzhou, China

**Keywords:** lipid-lowering, colon, rectum, neoplasm, cancer

## Abstract

**Background:**

While epidemiological studies have linked statin use to a reduced risk of advanced colorectal adenomas, its impact on colorectal cancer (CRC) risk in patients with inflammatory bowel disease (IBD) remains unclear. To our knowledge, no meta-analysis to date has specifically examined this association. Therefore, we conducted a systematic review and meta-analysis of the available observational studies to investigate the risk of CRC associated with statin use in IBD patients.

**Methods:**

We searched three databases for articles published in English before September 2024, focusing on the protective effects of statins against CRC in IBD patients. We calculated multivariate odds ratios (ORs) and their 95% confidence intervals (CIs) to assess this association. A random-effects meta-analysis was conducted using the generic inverse variance method.

**Results:**

The meta-analysis included 4 studies encompassing 22,250 IBD patients, 6,712 of whom were statin users. The methodological quality of three of the studies was deemed high. We found a significantly lower risk of CRC in statin users compared to non-users, with a pooled relative risk of 1.88 (95% CI 1.54–2.30). Sensitivity analyses confirmed the consistency of these findings.

**Conclusion:**

Statin use appears to be associated with a reduced risk of CRC in patients with IBD. However, given the limited number of studies available, further prospective research with large sample size is necessary to confirm the potential chemopreventive role of statins in this population.

## Introduction

Inflammatory bowel disease (IBD) encompasses a group of immune-mediated disorders that exhibit a relapsing–remitting course, including ulcerative colitis (UC), Crohn’s disease (CD), and IBD-unclassified colitis (IBD-U) (1). The global incidence of IBD is on the rise, presenting significant economic and social challenges to healthcare systems due to its high prevalence, early onset, and the requirement for lifelong treatment (2). IBD is associated with various complications such as anemia, stenosis, abscesses, and fistulas (3). Notably, colorectal cancer (CRC) represents a significant morbidity factor in IBD patients, with studies showing an estimated 2-fold increased risk compared to the general population (4). This risk is further elevated in patients with concomitant primary sclerosing cholangitis (5, 6). Recognizing the risk factors for CRC in IBD could aid in preventing the disease and guiding targeted interventions.

Statins are primarily prescribed to treat hypercholesterolemia and reduce cardiovascular morbidity and mortality (7). Beyond their lipid-lowering effects, statins have demonstrated anti-proliferative, anti-inflammatory, and anti-neoplastic properties in numerous preclinical studies (8–10). As a result, research has suggested a potential benefit of statins in reducing cancer incidence. In terms of CRC risk in the general population (11, 12), meta-analyses of observational studies have indicated a modest reduction in CRC risk, though randomized controlled trials have not shown significant benefits (13). Subsequently, several studies (14–17) have explored the link between statin use and CRC risk in patients with IBD. In the earliest study, Samadder et al. found no protective effect of statins against CRC. Similar results were reported in a U.S.-based hospital analysis. However, two larger population-based studies identified an inverse association between statin use and CRC. These findings raise the question of whether statin use is linked to a reduced risk of CRC in the IBD population.

In light of the growing prevalence of IBD and widespread use of statins, we conducted a systematic review and meta-analysis to evaluate the relationship between statin use and CRC risk among IBD patients. This review aimed to support the development of evidence-based clinical guidelines and inspire further research in this area.

## Methods

### Search strategy

This systematic review and meta-analysis was conducted in accordance with the PRISMA (Preferred Reporting Items for Systematic Reviews and Meta-Analyses) guidelines. We conducted a comprehensive search in PubMed and Embase databases for studies published up to September 10, 2024. Our search terms included combinations of “inflammatory bowel disease,” “IBD,” “Crohn’s disease,” “CD,” “ulcerative colitis,” “UC,” “statin(s),” “HMG-CoA reductase inhibitor(s),” “simvastatin,” “atorvastatin,” “pravastatin,” “fluvastatin,” “rosuvastatin,” “lovastatin,” and terms related to colorectal conditions such as “colon,” “rectal,” “colorectal,” along with “cancer,” “tumor,” “carcinoma,” and “neoplasm.” In addition, a manual search of the reference lists of the retrieved articles was conducted.

### Inclusion criteria

The inclusion criteria of our study followed the PICO (Population, Intervention, Comparison, Outcome) framework: (1) Population: patients diagnosed with IBD; (2) Intervention: statin use; (3) Comparison: non-use of statins; (4) Outcome: incidence of CRC. Randomized controlled trials (RCTs), cohort studies, and case–control studies were included, while case series, case reports, animal studies, editorials, and reviews were excluded.

### Data extraction and quality assessment

Data from the included studies were extracted and summarized in an Excel spreadsheet. Extracted information included the first author, publication year, study design, study location, subject characteristics (age and IBD type), methods for assessing IBD, the number of IBD patients exposed and unexposed to statins, CRC definitions, statistical adjustments for confounders, and study quality assessment.

Each article was independently evaluated using the Newcastle-Ottawa Quality Assessment Scale (NOS) (18), as recommended by the Cochrane Collaboration for assessing the quality of observational studies. A score higher than 7 points indicated a high-quality study.

### Statistical analysis

Statistical analyses were conducted using Stata 12.0 software (Stata Corp., College Station, TX, USA). Heterogeneity was assessed using the *I*^2^ statistic, with *I*^2^ > 50% indicating significant heterogeneity (19). In cases of significant heterogeneity, random-effects models were applied. The risks of CRC were expressed as odds ratios (ORs) with 95% confidence intervals (CI) for case–control studies, and as relative risks (RRs) or hazard ratios (HRs) with 95% CIs for cohort studies (20). ORs were treated as approximations of RRs or HRs due to the rarity of CRC in all populations. Fixed-effect models were used when no significant heterogeneity was found. A funnel plot was not generated because <10 studies were included (21). A 2-sided test was performed, and *p* < 0.05 was considered statistically significant.

## Results

### Search results

**Figure 1:**
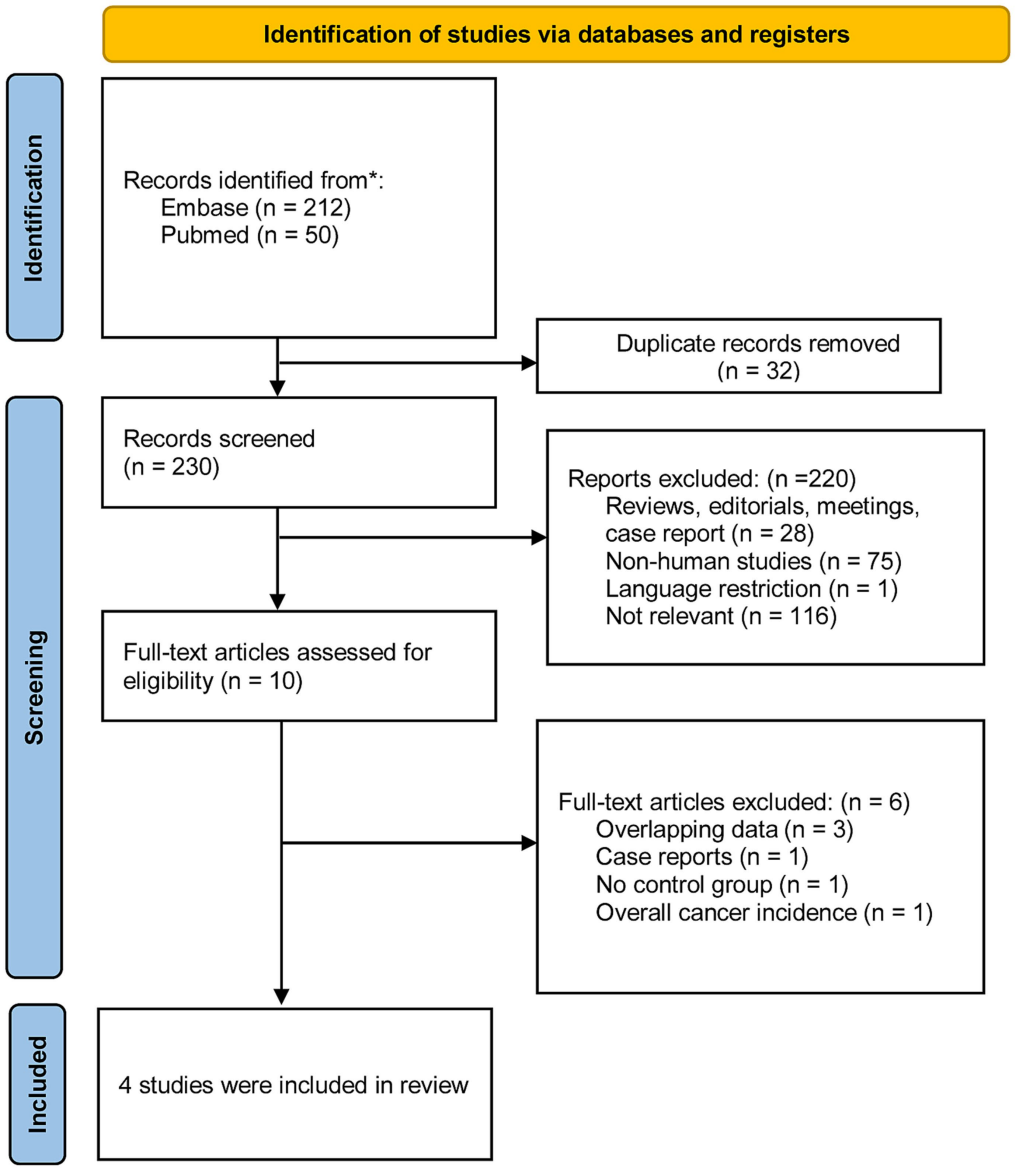
Flow chart of the search process and study selection.

We identified 262 studies from two databases using relevant keywords. After screening titles and abstracts, 32 duplicates and 220 studies were excluded, leaving 10 for full-text review. Six studies were excluded due to multiple reasons. Ultimately, four studies involving patients with IBD were included in our analysis. The selection process was further refined by addressing potential duplicates (due to shared databases and common co-authors) and by excluding case reports. Figure 1 provides a flow diagram outlining the literature search and selection process.

### Characteristics of included studies

Table 1 summarizes the characteristics of the included studies: two were conducted in North America, one in Europe, and one in Asia. These studies were published between 2011 and 2023, with sample sizes ranging from 60 to 11,001 participants. Of the four studies, three were cohort studies and one was a case–control study. Based on methodological quality assessments, three studies were deemed high quality, while one was categorized as low quality. Supplementary Tables S1, S2 provide detailed score breakdowns.

**Table 1:** Characteristics of the included studies.

Author, year	Location, setting	Study design/Study period	IBD assessment	Statin exposure assessment	No. of IBD	Age (year)	CRC assessment	Adjustment (Yes/no)	NOS
Samadder et al., 2011	Israel, population-based	Case-control, 1998–2004	Self-Reported	Questionnaire	60 (exposure 6) (no-exposure 54)	exposure 70 no-exposure 70	One pathologist confirmed diagnoses	Age, sex, ethnic group, presence or absence of sports participation, level of vegetable consumption, smoking status, and history of colorectal cancer in a first-degree relative	6
Ananthakrishnan et al., 2016	USA, population-based	Cohort, 1998–2010	ICD-9-CM	Electronic medical record system	1,1,001 (exposure 1,376) (no-exposure 9,625)	exposure 67 no-exposure 42	ICD-9	Age, sex, smoking, smoking status, ethnic group, drug use, primary sclerosing cholangitis, duration of inflammatory bowel disease	9
Shah et al., 2019	USA, hospital-based	Cohort, 2005–2016	ICD-9 and/or ICD-10	Medical record review	643 (exposure 57) (no-exposure 585)	exposure 59 no-exposure 39.4	ICD-9 and/or ICD-10	Statin use, age, sex, primary sclerosing cholangitis, duration of inflammatory bowel disease, mean inflammatory score, number of colonoscopies, thiopurine exposure and biologic exposure	6
Sun et al., 2023	Sweden, population-based	Cohort, 2006–2019	ICD-10	Electronic medical record system	10,546 (exposure 5,273) (no-exposure 5,273)	exposure 51 no-exposure 51	ICD-9	Propensity score matching	9

### Meta-analysis

**Figure 2:**
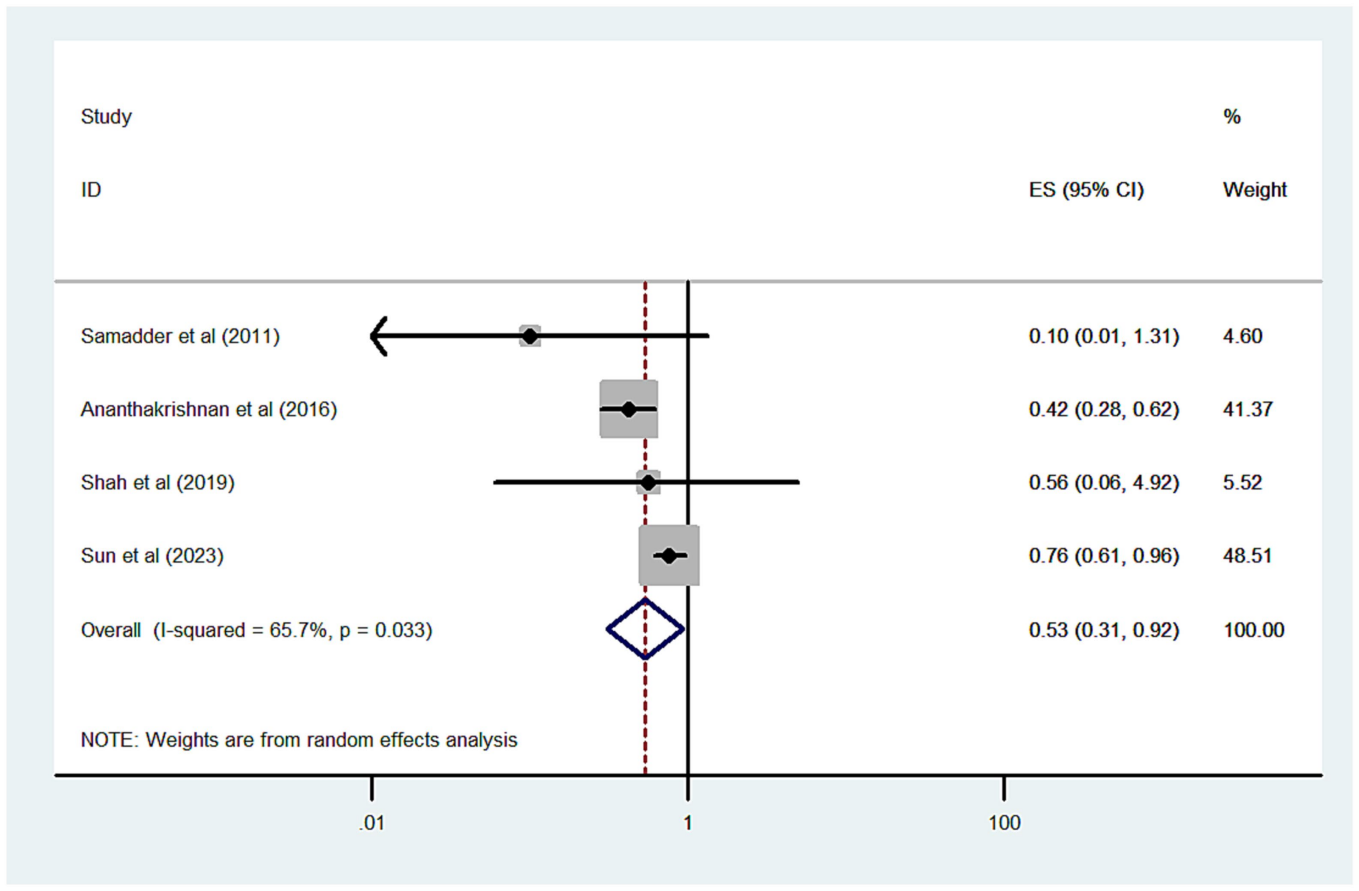
Forest plot of the overall risk of CRC in relation to exposure to statin among patients with IBD.

We analyzed 4 studies, comprising 6,712 statin-exposed and 15,537 unexposed patients with IBD, to assess the risk of CRC associated with statin use. The combined OR for CRC was 0.53 (95% CI 0.31–0.92; *p* = 0.024), indicating a protective effect, although there was significant heterogeneity (*I*^2^ = 65.7%) (Figure 2). When the analysis was limited to three high-quality cohort studies, the protective effect of statins on CRC risk remained consistent (OR = 0.58, 95% CI 0.35–0.97, *p* = 0.037; *I*^2^ = 69.1%). Sensitivity analysis showed no substantial change in the pooled risk estimates; the pooled ORs for CRC ranged from 0.41 to 0.58.

**Figure 3:**
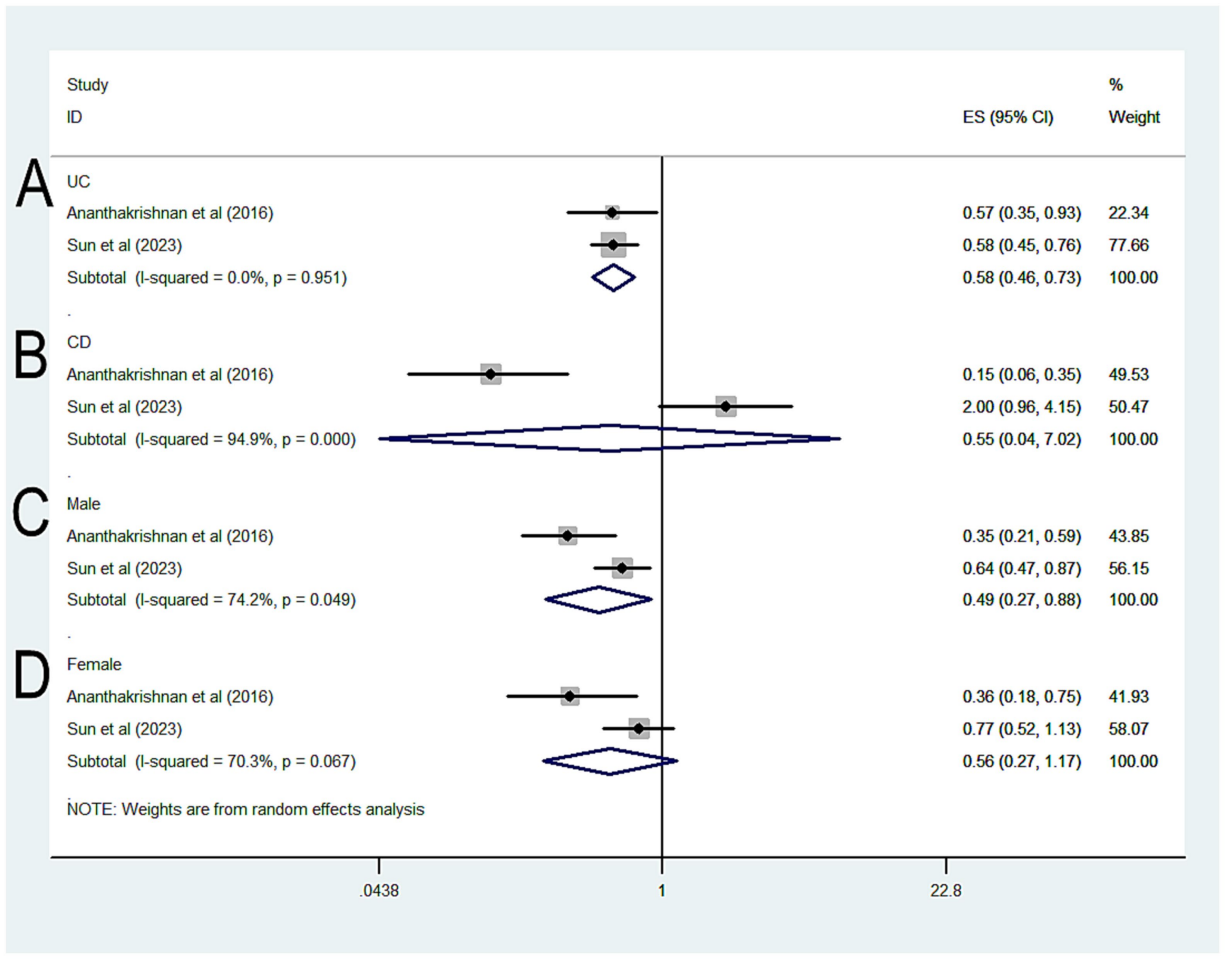
Forest plot of the overall risk of CRC in relation to exposure to statin among patients with IBD **(A)** UC; **(B)** CD; **(C)** Male; **(D)** Female.

Further analysis by type of IBD revealed that statin-exposed UC patients had a lower risk of CRC (OR = 0.58, 95% CI 0.46–0.73, *p* < 0.001; *I*^2^ = 0%) (Figure 3A), whereas no significant protective effect was observed in CD patients (OR = 0.55, 95% CI 0.04–7.02, *p* = 0.649; *I*^2^ = 94.9%) (Figure 3B). In sex-based analyses, a protective effect was seen in male IBD patients exposed to statins (OR = 0.49, 95% CI 0.27–0.88, *p* = 0.018; *I*^2^ = 74.2%) (Figure 3C), but not in female patients (OR = 0.56, 95% CI 0.17–1.17, *p* = 0.122; *I*^2^ = 70.3%) (Figure 3D).

## Discussion

This is the first meta-analysis to investigate the association between statin use and the risk of CRC in patients with IBD. Our findings suggest a significantly reduced risk of CRC in statin users compared to non-users, even after adjusting for potential confounding factors. However, due to the limited number of included studies, caution is warranted when interpreting these results.

The exact pharmacological mechanisms behind the antitumor effects of statins are not yet fully understood, but several explanations have been proposed. One of the primary mechanisms involves the inhibition of HMG-CoA reductase, the enzyme statins target to lower cholesterol. This inhibition reduces mevalonate synthesis, which is essential for cholesterol production (22). Interestingly, disruptions caused by statins in the mevalonate synthesis pathway inhibit cancer growth and lead to apoptotic cell death, and the depletion of cholesterol may inhibit cancer cell growth (23). Additionally, statins may inhibit the synthesis of isoprenoids, which are essential lipid attachments for intracellular signaling molecules, such as Rho, Rac, and Cdc42 (24). These related-proteins are overpresented in CRC and are associated with tumor invasion. Furthermore, statins may reduce the formation of aberrant crypt foci and polyps, and reduce tumor metastasis (25). Non-HMG-CoA-related effects of statins include anti-proliferative actions, regulation of cell adhesion, antioxidant properties, and anti-inflammatory effects (26). Finally, several reports have demonstrated that the use of statins may reshape the balance of gut microbiota in patients with hyperlipidemia and favors the growth of species whose metabolites may exert anti-inflammatory effects as *Bifidobacterium* (27). The anti-tumor effect of *Bifidobacterium* has been proved in *in vitro* and *in vivo* (28, 29).

Chronic intestinal inflammation is thought to play a critical role in the development of CRC (30). Persistent inflammation in IBD patients increases the risk of colorectal neoplasia and its long-term consequences, including CRC. A meta-analysis by Lutgens et al. (4) reported that IBD patients have a 70% higher risk of CRC compared to the general population. Since 2010, several meta-analyses (11, 12) based on observational studies have shown a lower CRC risk in the general population. Given the high incidence of CRC in IBD and the potential antitumor effects of statins, it is important to explore further the association between statin use and CRC risk in IBD patients.

Although the potential protective effects of statins on CRC in IBD are biologically plausible, the studies included in our meta-analysis reported inconsistent results, which is reflected in the significant clinical heterogeneity observed. A key source of this heterogeneity is the variation in sample sizes. Two studies reporting no protective effect of statins enrolled only 703 IBD patients and identified 44 CRC cases, making it reasonable to speculate that their findings may be influenced by small sample sizes. In contrast, the other 2 studies, with a combined total of 21,545 IBD patients, observed a protective role for statins in CRC prevention.

Previous meta-analysis (31) have demonstrated that colonoscopy can effectively reduce the incidence of CRC. In one of the included studies, Shah et al. explored the relationship between statin use and CRC in a cohort of IBD patients undergoing regular colorectal surveillance, which may have minimized the observed protective effect of statins. Additionally, the statin-exposed group in this study were older than the unexposed group, which is notable since CRC incidence increases with age. This uneven age distribution may have masked the effect of statins.

Other factors, such as exposure to chemopreventive agents, IBD medications, IBD severity, and the presence of primary sclerosing cholangitis, have also been associated with CRC development in IBD patients (32–34). However, the studies included in our meta-analysis adjusted for these confounding variables to varying degrees. The failure to account for these important factors in some studies may have influenced the strength and reliability of their conclusions.

This is the first and most comprehensive systematic review and meta-analysis to investigate the risk of CRC in statin users with IBD. However, our study has some limitations, particularly regarding unknown confounders. Given ethical and practical constraints, conducting RCTs to evaluate the chemopreventive effects of statins on CRC risk is not feasible. Future well-designed studies that account for additional variables, such as smoking status, chronic comorbidities, and other medication use, are needed to examine further this association. Second, another limitation is the high heterogeneity with respect to the characteristics of the included studies, and finding sources of heterogeneity is one of the most important goals of meta-analysis. In the present meta-analysis, this heterogeneity could not be explained by the sensitivity, or subgroup analyses based on type of IBD or gender. The existence of clinical heterogeneity would be the source of statistical heterogeneity in the results. One included study (14) observed a significant duration-dependent benefit. With respect to statin type, previous meta-analysis (35) showed a significant association between lipophilic statin use and CRC risk and a null association between hydrophilic statin use and CRC risk among the general population. However, the included studies provided limited data on the dose, duration, and type of statins used, preventing us from conducting further analyses about how these factors might influence CRC risk. Third, the number of eligible studies and the sample size of IBD patients and CRC cases were relatively small, which may have affected the accuracy of our findings. These results should be interpreted with caution, and more clinical and basic research is needed to confirm the potential protective effects of statins in IBD patients. Fourth, we were unable to assess the causal relationship between statin use and CRC risk, which would provide a deeper understanding of the association. Finally, most of the included studies were conducted in Western countries, limiting the generalizability of our findings to other populations.

In conclusion, our results suggest that statin use is associated with a reduced risk of CRC in patients with IBD, indicating potential for statins as a chemopreventive agent in this population. However, these findings need to be confirmed through larger, well-designed prospective studies.

## Data Availability

The original contributions presented in the study are included in the article/Supplementary material, further inquiries can be directed to the corresponding author.
