# Supplementary material for: Statin exposure and risk of colorectal cancer in patients with inflammatory bowel disease: a systematic review and meta-analysis
Source: Front Med (Lausanne). 2024 Nov 22;11:1507739. doi: 10.3389/fmed.2024.1507739 (PMC11624505; doi:10.3389/fmed.2024.1507739)
Supplement: Supplementary file 1 [file Data_Sheet_1.PDF]

Table S1 NOS for Assessment of Quality of Included Studies: Cohort Studies

Study	Selection				Comparability	Outcomes		
	Representativeness of exposed cohort?	Selection of the nonexposed cohort?	Ascertainment of exposure?	Demonstration that outcome of interest was not represent at the start of the study	Comparability of Cohort	Assessment of outcome	Was follow-up long enough for outcomes to occur	Adequacy of follow up of cohorts
Ananthakrishnan et al, 2016	★	★	★	★	★★	★	★	★
Shah et al, 2019	—	—	★	★	★	★	★	★
Sun et al, 2023	★	★	★	★	★★	★	★	★

Table S2 NOS for Assessment of Quality of Included Studies: Case-Control Studies

Study	Selection				Comparability		Exposure		
	Is the case definition adequate	Representativeness of cases	Selection of controls	Definition of controls	Study controls for age/sex	Study controls for at least 3 additional factors	Ascertainment of exposure	Same method of ascertainment of exposure	Nonresponse rate
Samadder et al, 2011	★	—	—	★	★	★	★	★	—
